# Synergetic Effects of Combined Nanomaterials for Biosensing Applications

**DOI:** 10.3390/s17051010

**Published:** 2017-05-03

**Authors:** Michael Holzinger, Alan Le Goff, Serge Cosnier

**Affiliations:** Department of Molecular Chemistry (DCM) UMR 5250, University Grenoble Alpes—CNRS, F-38000 Grenoble, France; alan.le-goff@univ-grenoble-alpes.fr (A.L.G.); Serge.Cosnier@univ-grenoble-alpes.fr (S.C.)

**Keywords:** nanomaterials, biosensors, hybrids, carbon, metals, semiconductors, energy transfer

## Abstract

Nanomaterials have become essential components for the development of biosensors since such nanosized compounds were shown to clearly increase the analytical performance. The improvements are mainly related to an increased surface area, thus providing an enhanced accessibility for the analyte, the compound to be detected, to the receptor unit, the sensing element. Nanomaterials can also add value to biosensor devices due to their intrinsic physical or chemical properties and can even act as transducers for the signal capture. Among the vast amount of examples where nanomaterials demonstrate their superiority to bulk materials, the combination of different nano-objects with different characteristics can create phenomena which contribute to new or improved signal capture setups. These phenomena and their utility in biosensor devices are summarized in a non-exhaustive way where the principles behind these synergetic effects are emphasized.

## 1. Introduction

The particularity of biosensors, compared to classic sensors, is that the sensing element, also called the receptor unit, is a biological entity or a bioinspired compound which confers an excellent selectivity towards the analyte to be detected. The unique specificity of such bioreceptors represents the main advantage within all sensor devices and the development of biosensors has become a huge research topic since highly complex solutions like blood can be analyzed for one specific target [1,2,3]. Biosensors are mainly used for the monitoring of diseases and are based on the recognition event of immune systems, viruses, bacteria, or cells, but also find utility for the detection of chemicals like blood sugar or pollutants [4,5]. One challenge is the signal capture during the biological recognition event [6,7].

Voltammetric biosensors rely on a redox process where the involved electron transfers are proportional to the analyte concentration [8]. For instance, the enzyme glucose oxidase (GOx) recognizes very specifically β-d-glucose, which is oxidized to gluconolactone. The reduced enzyme generally regenerates itself by reducing oxygen to hydrogen peroxide [9], a electroactive molecule which can be detected by the electrode. For immunosensing and the detection of DNA, more sophisticated setups are needed since an immune reaction or a hybridization of DNAs does not produce an electrochemical signal. For these cases, labeled secondary antibodies or DNA strands have to be involved after the recognition event where these labels will give the electrochemical signal. To avoid such supplemental time consuming preparation steps, electrochemical impedance spectroscopy (EIS) represents a very appropriate tool for immune and DNA sensors. EIS works with alternating currents (ACs) of small amplitude within a wide range of frequencies. The biorecognition event changes the sensing capacitance and interfacial electron transfer resistance of the electrode leading to a highly sensitive signal capture down to the femtomolar range [10,11].

Gravimetric biosensors are mostly piezoelectric devices where the detection of biological targets provokes a change of the resonance frequency related to the mass of the analyte [12,13]. One famous example is quartz crystal microbalance (QCM) but also micro- (or nano-) mechanical cantilever setups [14] are promising candidates for highly sensitive label free transduction techniques.

Most optical biosensors are based on a change in fluorescence or color during or after the recognition event [15]. As for electrochemical biosensors, some techniques need the use of supplemental labelling steps to introduce a photosensitive probe. Label-free optical detection can be achieved using surface plasmon resonance (SPR), which is a highly sensitive and quantitative transduction technique. The principle is based on the change of light-induced electron oscillations (surface plasmons) in the conduction band of metallic coatings (usually gold) when the dielectric constant of its environment changes [16]. This is the case, among others, for immune reactions or DNA hybridization where the recognition event changes the oscillation frequency which results in an angle change of the reflected light, its change of intensity, refractive index, or its phase [17,18].

The use of nanomaterials clearly already enhances the signal capture of all these transduction techniques used for biosensing thanks to their enhanced specific surface which allows the immobilization of an enhanced amount of bioreceptor units with an improved accessibility for the analytes. The advantages of different nanomaterials for biosensors are summarized in many review articles [19,20,21,22,23,24,25,26,27]. Here, we want to present some selected examples of synergetic effects achievable by combining different nanomaterials, thus enabling new or original transduction of biorecognition events.

## 2. Nanoparticles

Nanoparticles have become important components in biosensing devices since almost every material can be shaped into nanosized structures, thus conferring specific properties to the sensing element [28]. For instance, noble metal particles like silver and gold are famous for their localized resonant surface plasmons tremendously enhancing SPR or Raman signals [29,30,31]. These and other materials like platinum nanoparticles [32], or metal oxide nanoparticles [33] also provide improvements in catalysis and conductivity in electrochemical biosensors, while original setups were developed using magnetic nanoparticles [34]. Since many of these materials shows synergetic effects with other nano-objects, several examples will be described in more detail in the following sections. The most exploited synergetic effect between nanostructured materials is based on non-radiative energy transfer using upconverting nanoparticles (UCNPs) and quantum dots (QDs).

### 2.1. Upconverting Nanoparticles

**Figure 1:**
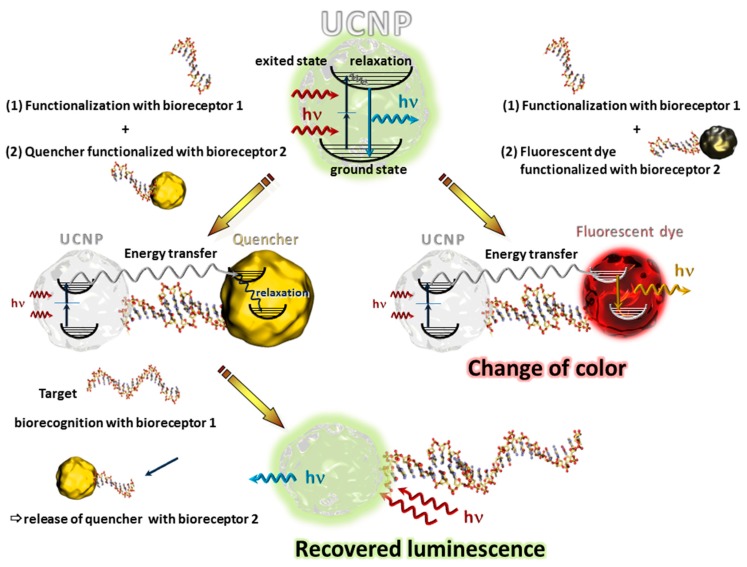
Schematic presentation of an UCNP and its anti-stokes type emission (top) and their functioning as bioanalytical transducer using a nanosized quencher (left) or a fluorescent dye (right).

UCNPs have the capacity to absorb several photons in the infrared range and to convert this absorbed energy into an emission in the visible range via a nonlinear optical process [35]. Contrary to common multiphoton absorption materials, these nanoparticles do not need high excitation densities for efficient anti-Stokes type emission. The phenomenon of high wavelength absorption and low wavelength emission strongly depends on the ion-ion distance of a dopant (mostly lanthanides) in a host material (generally Na^+^ or Ca^2+^ fluorides). The confinement of the lanthanides in the matrix also determines the color of emitted light [36,37]. UCNPs became promising alternatives to other fluorescent labels for biosensing applications since they have very low background emissions and the high excitation wavelength does not provoke luminescence or absorption effects with other components of the biosensor [38,39]. UCNPs can be used as simple labels but more interesting are transduction principles based on non-radiative resonance energy transfer (RET) from the exited UCNPs to an acceptor where it should be emphasized that lanthanides are luminescent and not fluorescent and the RET is called luminescent resonant energy transfer (LRET) contrary to fluorescent (or Förster) resonant energy transfer (FRET) using fluorescent dyes [40]. The acceptor also plays a crucial role for this type of transduction because RET can only be achieved at corresponding quantum yields and cross sections, donor-acceptor distances, and their spectral emission and absorption overlap [41]. Furthermore, when the acceptor is a fluorescent probe, it can be excited by the UCNP leading to a change of the emission spectrum or simply to a change of the color of emitted light, or, when the acceptor is not a fluorophore, this results in the quenching of the emission of UCNPs [42] as illustrated in Figure 1.

Many approaches have been proposed relying on a quenching effect. For instance, Wang et al. demonstrated LRET between biotin-functionalized UCNPs and biotin-functionalized gold nanoparticles in the presence of avidin, which served here as the analyte which brings the two nano-objects in close contact leading to a linear reduction of the intensity of emitted light as a function of the avidin concentration [42]. A more sophisticated strategy was applied for the detection of thrombin in human plasma [43]. The specific thrombin aptamer was attached to the UCNPs while its luminescence is quenched in presence of carbon nanoparticles which form weak interactions with the aptamer. When the analyte is added, the carbon nanoparticles are released due to the stronger interaction between the aptamer and thrombin leading to a linear luminescence increase. A similar transduction principle was chosen by Zhang et al. who modified UCNPs with concanavalin A which interacts with saccharides. As quencher, chitosan-labeled graphene oxide was chosen and, due to the concanavalin A-chitosan interaction, the two components are assembled in close contact leading to the extinction of light. Then, glucose was used as analyte which forms stronger interaction with concanavalin A than chitosan leading to a glucose concentration dependent increase of emitted light [44].

The possibility to transfer upconverted energy to fluorophores thus changing the emission band after a biorecognition event has been extensively studied by Mattsson et al. [45]. For the proof of concept the biotin-streptavidin binding event was also used here as model recognition system. Streptavidin-modified UCNPs were attached to biotin-modified quantum dots leading to a change of the emission wavelength (the one for the quantum dots). In the presence of free biotin, mixed emissions or only the UCNPs’ luminescence could be observed. The possibility to calibrate the intensities of different wavelengths might represent a promising platform for multiplex biosensing.

However, one drawback of UCNPs for biosensing applications has to be noted. All mentioned examples need an excitation wavelength of 980 nm which is right in the absorption band of water and heats the sample. This inconvenience can be overcome by doping UCNPs with neodymium ions lowering the excitation wavelength to 808 nm. Sample heating can thus be avoided which is of particular importance for in vivo bioimaging [46] and this approach also shows advantages in the monitoring of enzymatic reactions [47].

### 2.2. Quantum Dots

**Figure 2:**
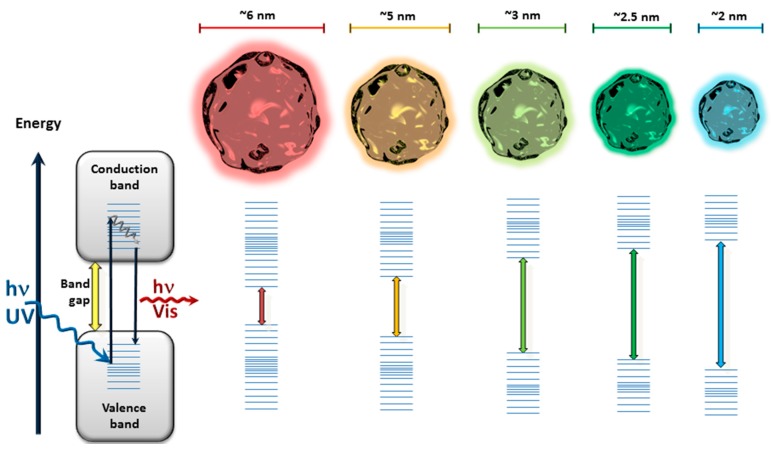
Illustration of QDs with different sizes and the related band gaps leading to different emission wavelengths after excitation with UV light.

QDs have become almost the nanomaterial of choice for fluorescence-based transduction in bioanalytics. QDs are luminescent semiconducting nanocrystals principally based on cadmium chalcogenides [48,49,50]. Most of them are available as core-shell particles coated with ZnS or CdS for enhanced quantum yields and photostability [51,52]. They can absorb in a large wavelength range but have a narrow emission spectrum which is dependent on the particle size [53] (Figure 2).

**Figure 3:**
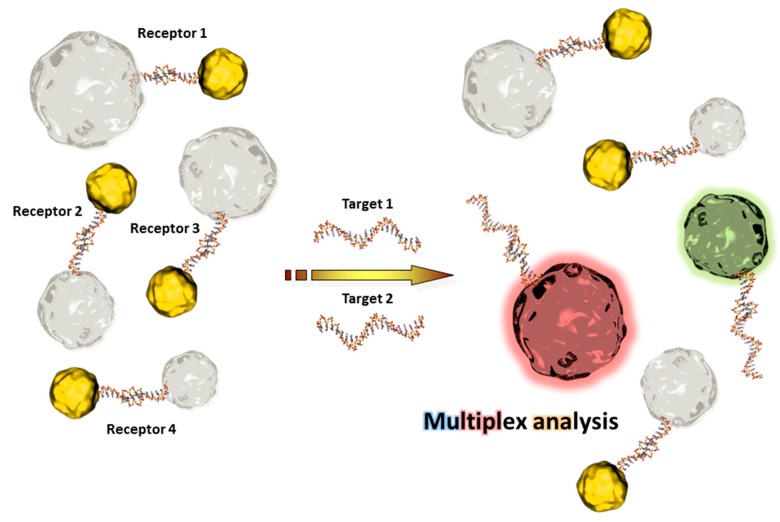
Scheme of a multiplex sensing principle using QDs and quenchers.

The availability of QDs with different emission wavelengths has made them promising candidates for multiplexed analysis [54,55,56], as depicted in Figure 3. Furthermore, a final coating of QDs allows efficient functionalization with bioreceptor units and can overcome possible toxicity issues [57].

QDs are, as UCNPs, excellent optical transducers in combination with other nanomaterials. The principle is mostly based on the release of a quencher after the recognition event and the recovery of fluorescence. This strategy is particularly efficient for aptamer- and DNA sensors [58,59]. As a representative example, the assembly of a QD-labeled receptor DNA with a shorter corresponding DNA tagged with a gold nanoparticle is cut by the (longer) analyte DNA due to its higher hybridization kinetics. The gold nanoparticle is released and the QDs start to emit light again where the intensity is proportional to the analyte concentration [60,61] (Figure 3).

Gold nanoparticles are not only used as non-radiative quenchers, but can also act as antennas for increased fluorescence of QDs due to their high plasmonic behavior. When gold nanoparticles are localized at around 30 nm to the QD surface, the gold nanoparticle provokes an increase of the excitation rates of the QDs and hence the intensity of the fluorescence [62]. Further non-radiative energy transfer leading to QD fluorescence can be achieved using emitting protein labels which eliminate the need of external excitation light source [63]. There are also charge transfer quenching and chemiluminescence resonance energy transfer phenomena [64] to complete the most common applied principles of FRET-based biosensing using QDs [65,66,67].

QDs were also combined with magnetic nanoparticles for improved biodetection [68]. The magnetic nanoparticles are used for the separation of biological analytes in complex media like blood or any type of body fluids. In detail, receptor unit-modified magnetic nanoparticles are introduced in the analyte solution and interact specifically with the target molecule. The particles then migrate in a magnetic field until settling to form a deposit. The remaining solution can then be removed and the deposit can even be rinsed to eliminate any trapped species. QDs functionalized with a secondary receptor unit interact with the analyte on the magnetic particles and can quantify the detection via the intensity of the QD emission. A more sophisticated setup was proposed by Kurt et al. [69]. QDs and UCNPs, functionalized with different aptamers for different targets, served as recognition and transduction element in combination with magnetic nanoparticles modified with corresponding short DNA strands. The principle is based on the affinity interaction between aptamers and DNA linking weakly the magnetic nanoparticles with QDs and UCNPs which can then be separated from the solution in a magnetic field. In presence of the analytes (here the pathogens *Salmonella typhimurium* and *Staphylococcus aureus*), the DNA-aptamer link is broken by the competitive interaction with the target and the luminescent particles remain in solution after applying a magnetic field. The *Salmonella typhimurium*-UCNP and *Staphylococcus aureus*-QD assemblies can then be removed by washing thus leading to reduced intensities of emitted light. The authors observed a linear decrease of luminescence intensity with the analyte concentration. For the multiplex sensing setup, the remaining *Staphylococcus aureus* specific QDs and *Salmonella typhimurium* specific UCNPs are exited at 325 nm and 980 nm. UCNP excitation at 980 nm cannot excite the QDs since the energy of photons is below the band gap of the QDs. The authors observed negligible excitation of the QDs by the emission of UCNPs at 470 nm and could also exclude FRET. This setup might be a promising strategy for facilitated multiplex analysis but will need materials with narrower excitation and emission lines to prevent overlap or crosstalk effects.

**Figure 4:**
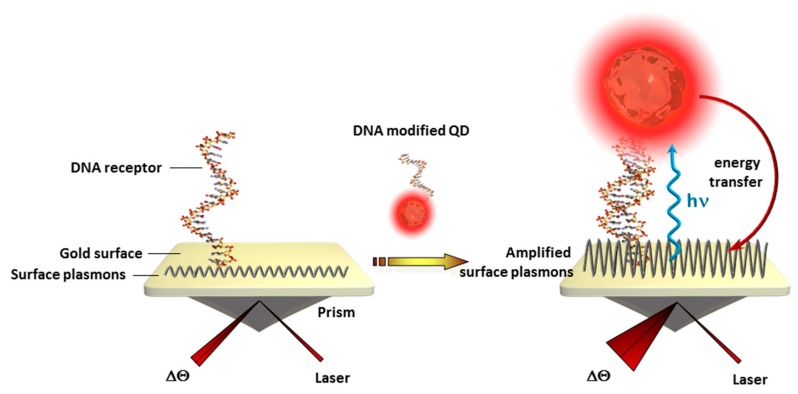
Schematic illustration of SPR signal amplification after the biorecognition event using QD labeled biomarkers.

Besides FRET with other nanomaterials, QDs can also interact with propagating surface plasmons of gold surfaces leading to light emission of the QDs or the light induced excited state of QDs are transferred to the surface plasmons [70] as illustrated in Figure 4. The second effect led to clear signal enhancements in SPR setups where a 25-fold increase was observed for ss-DNA and a 50-fold increase could be obtained with prostate-specific antigens compared to bare gold surfaces. [71].

QDs also show remarkable properties in electrochemical biosensing devices in combination with CNTs [72]. The distribution of these semiconductors within a CNT composite matrix forms domains with altered conductivities behaving like a microelectrode array. This phenomenon results in a clear reduction of the double layer capacitance and thus to an improved noise signal ratio. This setup was validated for the detection of hydrogen peroxide and ascorbic acid.

## 3. Carbon Nanomaterials

Carbon is a privileged material for biosensing applications, especially for electrochemical transduction due to its excellent conductivity and biocompatibility [73]. Carbon appears in many different allotropes based on graphite (sp^2^), diamond (sp^3^) and intermittent sp^2^-sp^3^ hybridized macroscopic structures generally called amorphous carbon, from which vast amounts of substructures can be synthesized [74]. For electrochemical biosensing, glassy carbon, doped diamond, and graphite are standard materials for electrodes [75]. Their nanostructured part in the form of carbon nanotubes [76], fullerenes [77], or graphene [78] partly became the material of choice for improved performances of bioanalytical devices [79]. More recently, fluorescent carbon nanodots have attracted attention as non-toxic alternatives to quantum dots for optical biosensing and bioimaging [80]. Efficient functionalization techniques were established for carbon nanomaterials which allow the formation of bioassemblies and to combine the beneficial properties with those of other nanosized materials [81]. This also allows reproducible processing and shaping to obtain the desired properties. An elegant way to assemble different materials is the formation of composites. As an example for electrochemical transduction, carbon paste electrodes provide unlimited possibilities to combine any type of carbon material with (nanosized) fillers conferring improved performances to the biosensor device. Selected examples and procedures of customized carbon paste-based biosensors were summarized by Muñoz et al. [82]. The following section presents some further examples of successful combinations of nanostructured carbon allotropes with other materials with synergetic effects for enhanced biosensor performance.

### 3.1. Graphene

Graphene has become a fashionable material for biosensing because it is considered less toxic than CNTs [83,84]. Even though graphene is per definition not a nanomaterial [85], it is worth summarizing some examples of its synergetic effects with other nano-objects since it belongs to the rich carbon allotrope family.

For electrochemical transduction, graphite-based layered materials are used in bulk form but are also often called graphene or graphene-like 2D materials [86]. Obtained after mechanic exfoliation [87,88], chemical oxidation of graphite [89] and/or subsequent reduction [90], these carbon materials are represented in many biosensor application examples [91] such as electrochemical immunosensors [92] or enzymatic biosensors [93]. In terms of synergetic hybrid materials, and similar to CNT hybrids, many different metal nanoparticles like gold [94], platinum [95,96,97,98,99], or palladium [100], or metal oxide nanoparticles [101] clearly improved the sensing performance when combined with graphene and graphene-like 2D materials.

For optical transduction, graphene materials can also act as non-radiant energy acceptors in FRET-based biosensors using organic dye- [102] or quantum dot [103] -labeled bioreceptors like DNAs, aptamers, or proteins [104]. Graphene oxide itself shows photoluminescence and can act as both, energy donor and energy acceptor [105] with excellent quenching efficiency [106].

In particular, graphene can interact with DNA or oligonucleotide receptors in a non-covalent and reversible manner, contrary to CNTs, and these dye-labeled receptors desorb after the recognition, recovering the fluorescence of the labels. This principle could even be applied for a multiplexed colorimetric DNA sensor [107]. Weak interactions with graphene oxide can also be obtained with antibodies labeled with QDs, which were exploited for the detection of the model pathogen *E. coli* [108]. The fluorescence of a corresponding antibody-modified QD is quenched in the presence of graphene oxide and recovered after the recognition event with the bacteria. This setup was successfully applied on nanocellulose-based papers for fluorescence biosensing using QDs and UCNPs [109]. Furthermore, such papers also provide an excellent platform for colorimetric sensing of biorelevant chemicals when functionalized with silver or gold nanoparticles.

**Figure 5:**
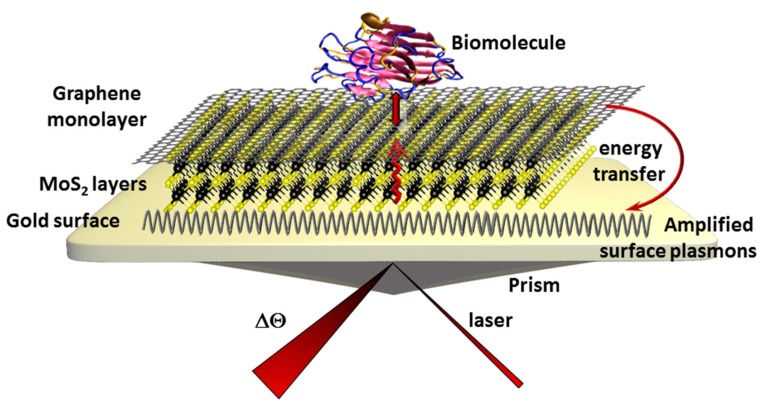
Principle of improved SPR signals after adsorption of a biomolecules using MoS_2_ as intermittent layer between monolayer graphene and the gold surface.

Furthermore, real monolayer graphene provides impressive beneficial properties in resonant plasmon transduction techniques [110]. Firstly predicted by theoretical models, these plasmonic properties of a single layer of graphene can interact with the surface plasmons of gold surfaces thus significantly amplifying the optical sensitivity of surface plasmon resonance (SPR) sensors [111]. By excitation in the visible light range [112] the propagation constant of surface plasmon polaritons (SPPs) is changed and the refractive index response in particular is amplified [113]. An almost two- fold increase of the SPR signal could be obtained just in presence of a graphene monolayer on gold which was validated in a highly sensitive anti-cholera toxin SPR sensor [114]. This phenomenon can in theory be further optimized using intermittent MoS_2_ layers [115]. Due to the improved optical absorption efficiency, the graphene-MoS_2_ layer can transfer this energy to the underlying gold layer thus further exciting and amplifying the resonant surface plasmons (Figure 5).

The authors calculated an up to 500-fold increase of phase sensitivity of the SPR signal with theoretic models when a biomolecule is adsorbed on the graphene layer via π-π stacking interactions. The authors unfortunately did not precise which biomolecule these calculations were based on.

### 3.2. Carbon Nanotubes

Carbon nanotubes (CNTs) can be seen as seamlessly rolled up graphene with one to up to hundreds of concentric wall layers and provide excellent 1D conductivity and high aspect ratios which form entangled porous structures in bulk, drastically increasing the accessible surface area of electrodes [116,117,118]. Furthermore, efficient and reliable functionalization methods were developed for the immobilization of bioreceptor units on CNTs without altering the biological activity [119].

CNTs were confined with Pt nanoparticles in a Nafion matrix for improved DNA sensing using daunomycin, a redox active compound which intercalates hybridized DNAs [120]. Single stranded receptor DNA was immobilized on this composite and was exposed to different concentrations of the analyte, the corresponding ssDNA. Since daunomycin only intercalates after the recognition event, the differential pulse voltammetric signal increased for the electrocatalytic reduction of the electrochemical probe. The combination of the enhanced specific surface area of CNTs and the catalytic properties of Pt led to clearly improved performances compared to setups using the individual compounds.

CNT-gold nanoparticle (AuNPs) assemblies clearly improve electrochemical transduction due to enhanced electron transfer rates between an enzymatically generated substrate and the AuNPs-CNT composites. A highly sensitive choline biosensor was developed based on choline oxidase modified AuNPs-CNT electrodes [121]. After the enzyme-catalyzed oxidation of choline, hydrogen peroxide is released which is finally oxidized on the electrocatalytic nanocomposite electrode. Beside the beneficial effect of AuNP-CNT assemblies for electrochemical biosensors [122,123], other metal or metal oxide nanoparticles showed improved biosensing performances when combined with carbon nanotubes. Most examples describe the improved electrocatalytic oxidation of enzymatically generated H_2_O_2_ using cobalt hexacyanoferrate nanoparticles [124], Pt nanoparticles [125], or ZnO nanoparticles [126] while for this example CNT-graphene hybrids were used. There are many further examples of using different nanomaterials in combination with CNTs which are summarized in reference [127].

### 3.3. C_60_ Fullerenes and Carbon Dots

C_60_ is the first fully characterized carbon nano-object and is classified as a 0D material. Its molecular structure is composed of 12 five-membered rings surrounded by a total of 20 six-membered rings and it obeys perfectly Euler’s rule [128]. The particular electrochemical properties of C_60_ [129] evoked much attention for its possible application as a redox mediator in enzymatic biosensors [130]. C_60_ also showed remarkably enhancements of the specific surface of electrodes and was used as a building block for original nanoscaffolds [131]. An electrochemical aptasensor was reported where an electrode surface is modified with onion-like mesoporous graphene sheets, gold nanoparticles, and a first aptamer receptor. Prussian Blue-modified gold nanoparticles were adsorbed on amine-functionalized C_60_ together with a second aptamer receptor and alkaline phosphatase as label. After the recognition event of the model target platelet-derived growth factor B-chain, and the formation of the sandwich structure, the immobilized enzyme label hydrolyzes ascorbic acid phosphate to ascorbic acid which is then oxidized on the Prussian Blue/gold nanoparticles/C_60_ electrode. Further examples describe the combination of C_60_ with mostly gold or platinum nanoparticles for improved electrochemical [132] or gravimetric [133] immunosensors, or an electrochemiluminescent aptasensor [134] but all of them rely on the electrochemical behavior of C_60_ or the capability to enhance the surface area and not on synergetic phenomena between these nano-objects.

**Figure 6:**
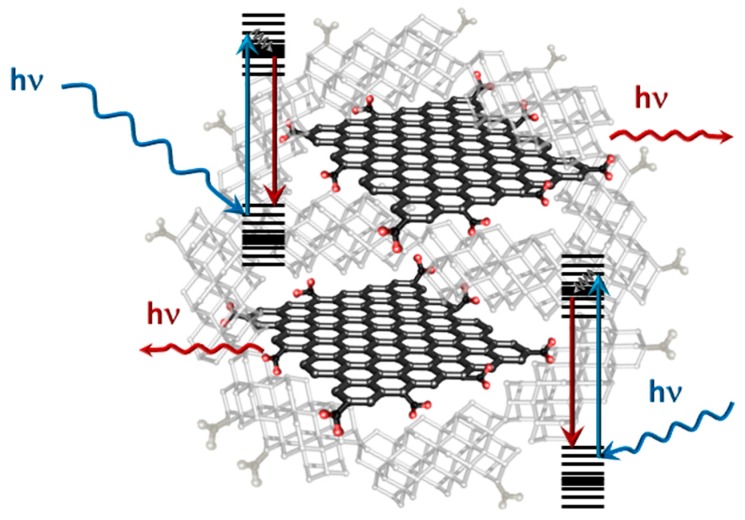
Sketch of a carbon QD with its defined sp^2^ domains isolated and surrounded with diamond- like carbon which is highly oxidized on the surface of the particle.

Carbon dots or carbon quantum dots, sometimes also called graphene quantum dots, can be considered further 0D carbon nanomaterials. Accidently discovered as a side product during arc discharge synthesis of single walled CNTs [135], these carbon QDs are promising candidates to replace heavy metal semiconductor QDs since they are still considered as a non-toxic carbon material with very satisfying quantum yields where even upconverting nanoparticles could be isolated and studied [136,137]. The fluorescence phenomenon is related to isolated domains of conjugated sp^2^ carbon surrounded by diamond like sp^3^ carbon [138] as depicted in Figure 6.

The fluorescence is also influenced by the mostly carboxylated surface which confers carbon QDs excellent solubility, but also strong pH-dependent fluorescence emission [139]. Even when great progress was achieved in the synthesis and isolation of carbon QDs with specific properties, the controlled synthesis of defined domain distribution and surface functionalities leading to distinguished absorption and emission spectra, as it is the case for semiconductor QDs, remains a challenge [140,141]. In terms of biosensing applications, carbon QDs show similar performances as semiconductor QDs concerning FRET-based biosensing and as fluorescence labels [80]. Efficient FRET between gold nanoparticles and carbon QDs could be achieved when each nano-object is modified with a corresponding antibody-antigen system [142]. In the presence of the analyte, in this example an organic pollutant, these assemblies are broken, leading to the recovery of fluorescence. Based on the same principle, a DNA sensor was proposed using assemblies of carbon QDs and fluorescence dye quenching each other whereby fluorescence reappears after the recognition event [143]. It would be interesting to study the efficiency and performances of carbon QDs and semiconductor QDs under identical condition to gain insight into the real potential of carbon QDs. It might be assumed that such studies will be reported in the near future.

## 4. Conclusions

Synergetic effects of different nanoparticles became promising tools for highly sensitive biodetection applications where the FRET effect is at the moment the most promising example where QDs were shown to be particularly versatile when combined with nanosized acceptors. Carbon QDs or UCNPs are promising candidates with lower toxicity issues to one day replace semiconductor QDs. In regards of the steady growing availability of different nanomaterials with different properties revealing new phenomena when in contact, other original electronic, electrochemical, or magnetic transduction methods can be developed. There remains one famous example of a nano-object with synergetic properties to which a section was not dedicated in this review: gold nanoparticles. This is simply due to the fact that nanosized gold was mentioned with almost all discussed materials and to avoid repetition, a separate discussion about gold-hybrids was intentionally omitted. However, for more information, examples of the beneficial properties of gold nanoparticle hybrids for biosensing and diagnostics are summarized in reference [144].
